# Supplementary figures and images for: Expression of Trefoil Factor 1 (TFF1) in Cancer: A Tissue Microarray Study Involving 18,878 Tumors
Source: Diagnostics (Basel). 2024 Sep 28;14(19):2157. doi: 10.3390/diagnostics14192157 (PMC11475926; doi:10.3390/diagnostics14192157)

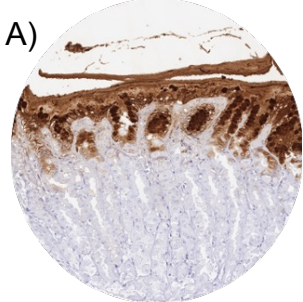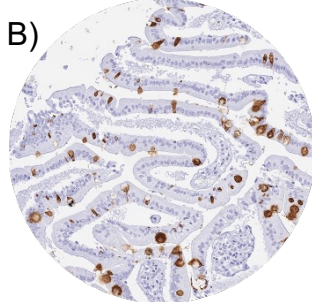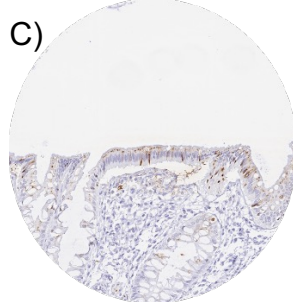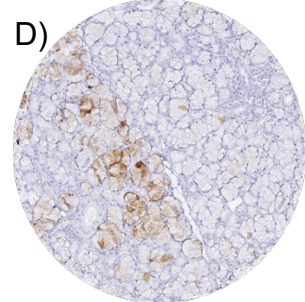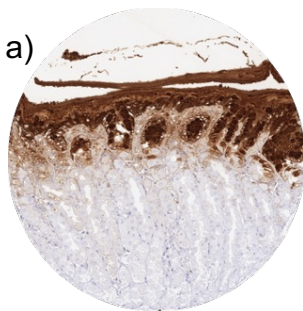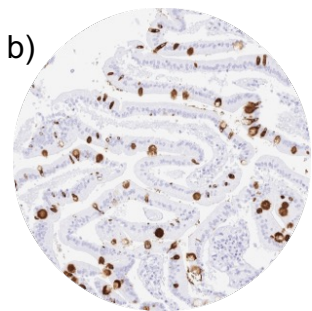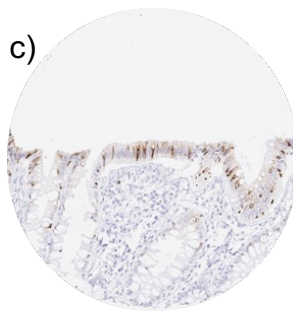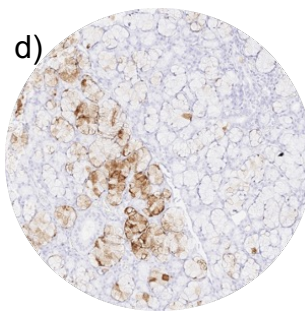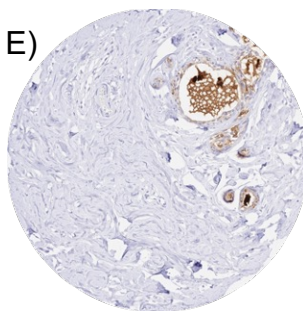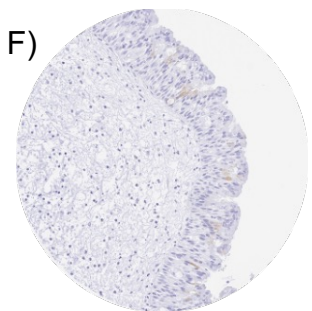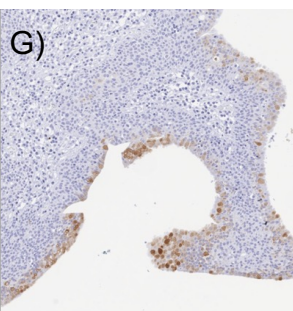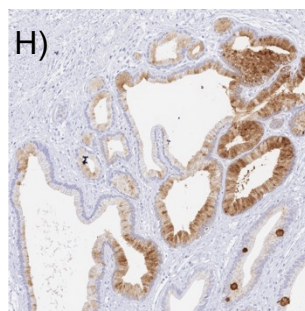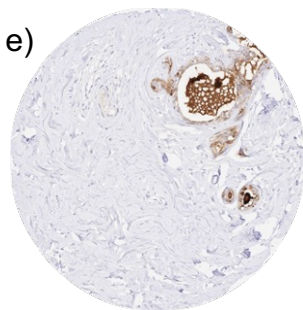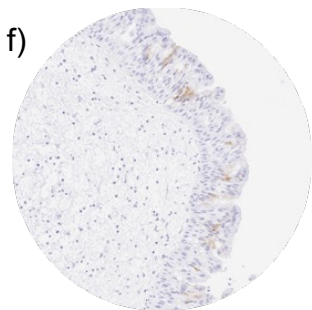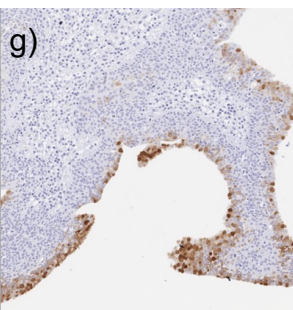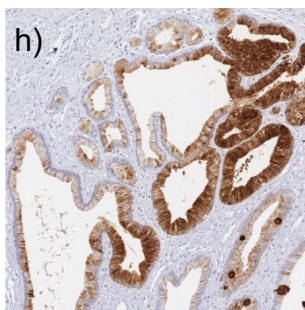

Supplement: Supplementary file 1 [file diagnostics-14-02157-s001.zip › Supplementary Figure S1.pdf]
